# Supplementary figures and images for: The dispensability of 14-3-3 proteins for the regulation of human cardiac sodium channel Nav1.5
Source: PLoS One. 2024 Mar 7;19(3):e0298820. doi: 10.1371/journal.pone.0298820 (PMC10919853; doi:10.1371/journal.pone.0298820)

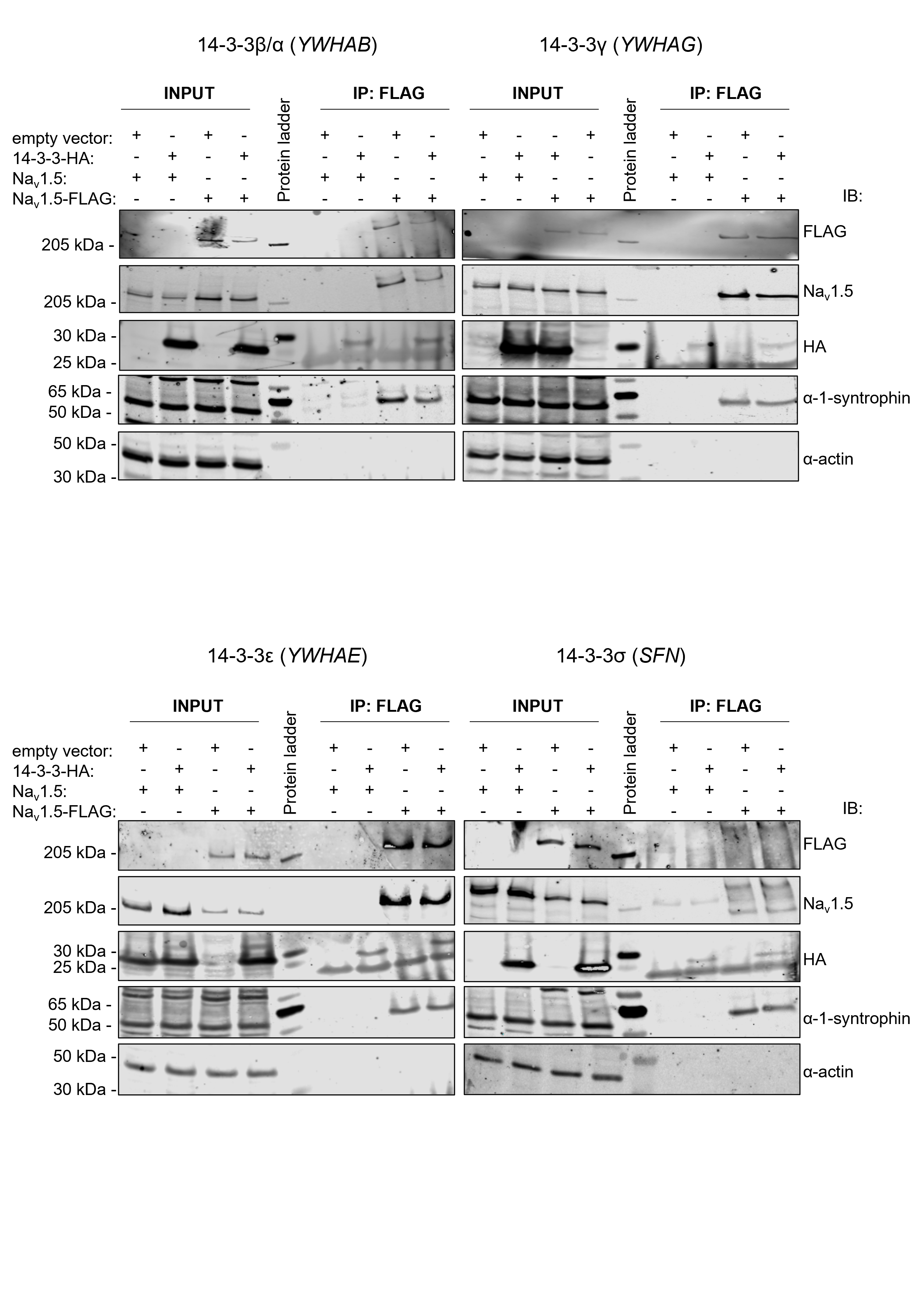

Supplement: S1 Fig — 48 hours after transient overexpression of 14-3-3 β/α (encoded by YWHAB), 14-3-3 γ (encoded by YWHAG), 14-3-3 ε (encoded by YWHAE), 14-3-3 σ (encoded by SFN) in tsA201 expressing Nav1.5 or Nav1.5-FLAG. Endogenous α-1-syntrophin was used as a positive control for co-immunoprecipitation with Nav1.5, and α-actin as a negative control. (TIF) [file pone.0298820.s001.tif]

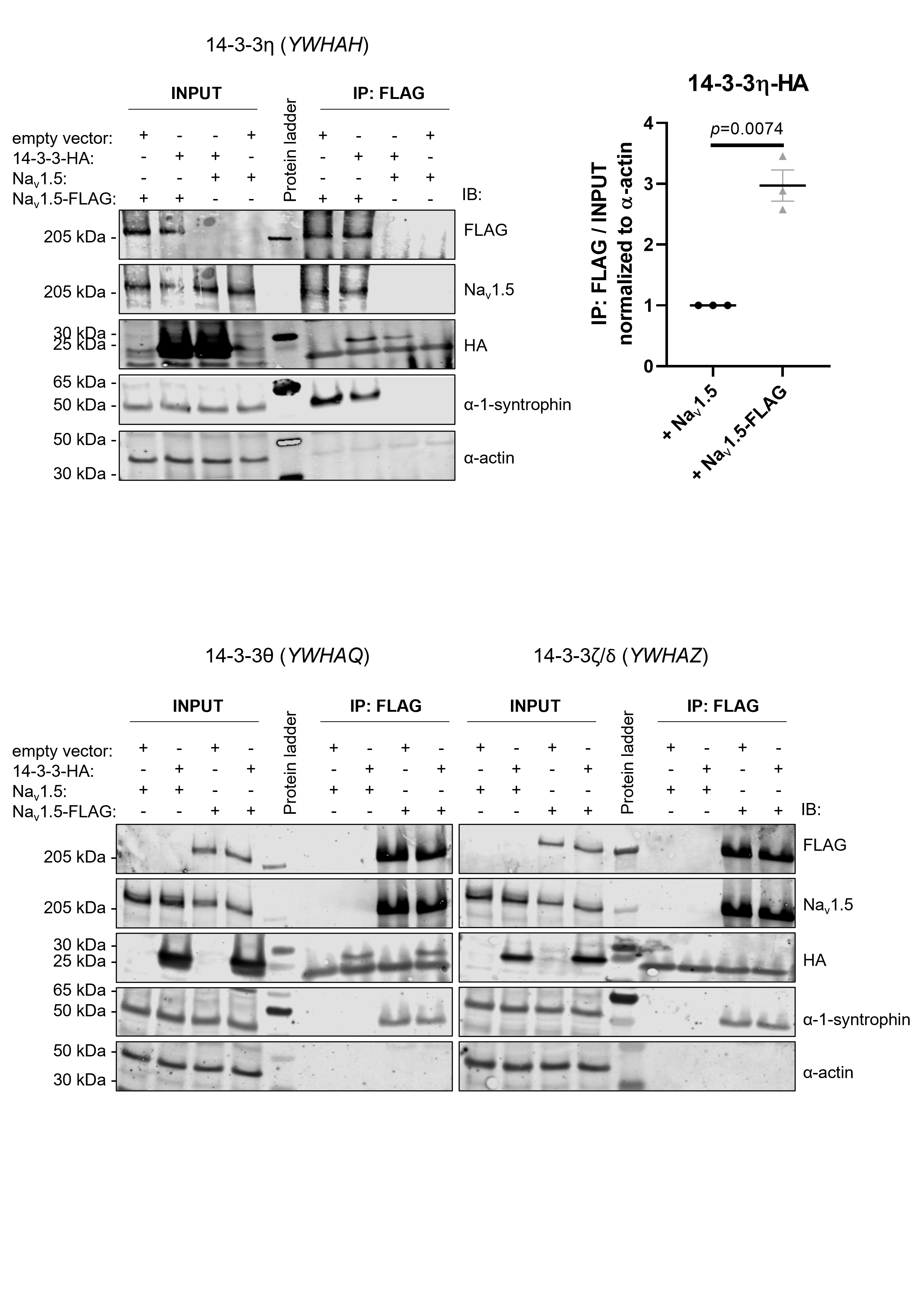

Supplement: S2 Fig — 48 hours after transient overexpression of 14-3-3η (encoded by YWHAH), 14-3-3θ (encoded by YWHAQ) and 14-3-3ζ/δ (encoded by YWHAZ) in tsA201 expressing Nav1.5 or Nav1.5-FLAG. Endogenous α-1-syntrophin was used as a positive control for co-immunoprecipitation with Nav1.5, and α-actin as a negative control. Intensity of Nav1.5-immunoprecipitated 14-3-3η-HA was normalized to the intensity of the total 14-3-3η-HA divided by the intensity of α-actin. Data are presented as mean ± SEM from three biological replicates and are normalized to the control condition (“+ Nav1.5”). Individual p-value, calculated with one-sample two-tailed t-test with hypothetical mean value = 1, is indicated in the panel. (TIF) [file pone.0298820.s002.tif]

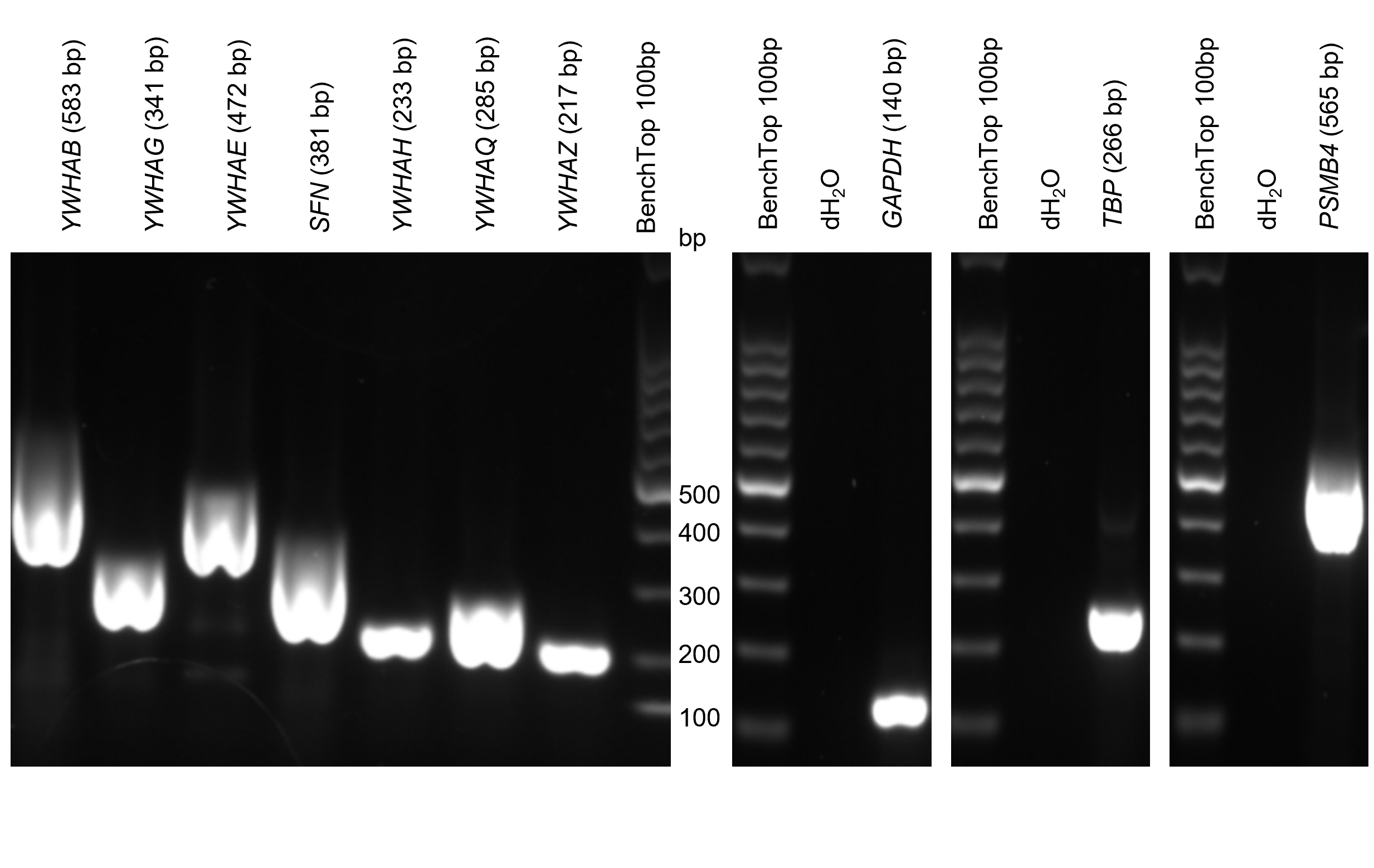

Supplement: S3 Fig — Conventional RT-PCR on tsA201 WT cells showing endogenous transcripts of all seven 14-3-3 isoforms (14-3-3β/α encoded by YWHAB, 14-3-3γ encoded by YWHAG, 14-3-3ε encoded by YWHAE, 14-3-3σ encoded by SFN, 14-3-3η encoded by YWHAH, 14-3-3θ encoded by YWHAQ, and 14-3-3ζ/δ encoded by YWHAZ) as well as housekeeping genes (glyceraldehyde-3-phosphate dehydrogenase encoded by GAPDH, TATA-binding protein encoded by TBP, and proteasome 20S subunit β4 encoded by PSMB4) with their expected amplicon sizes. (TIF) [file pone.0298820.s003.tif]

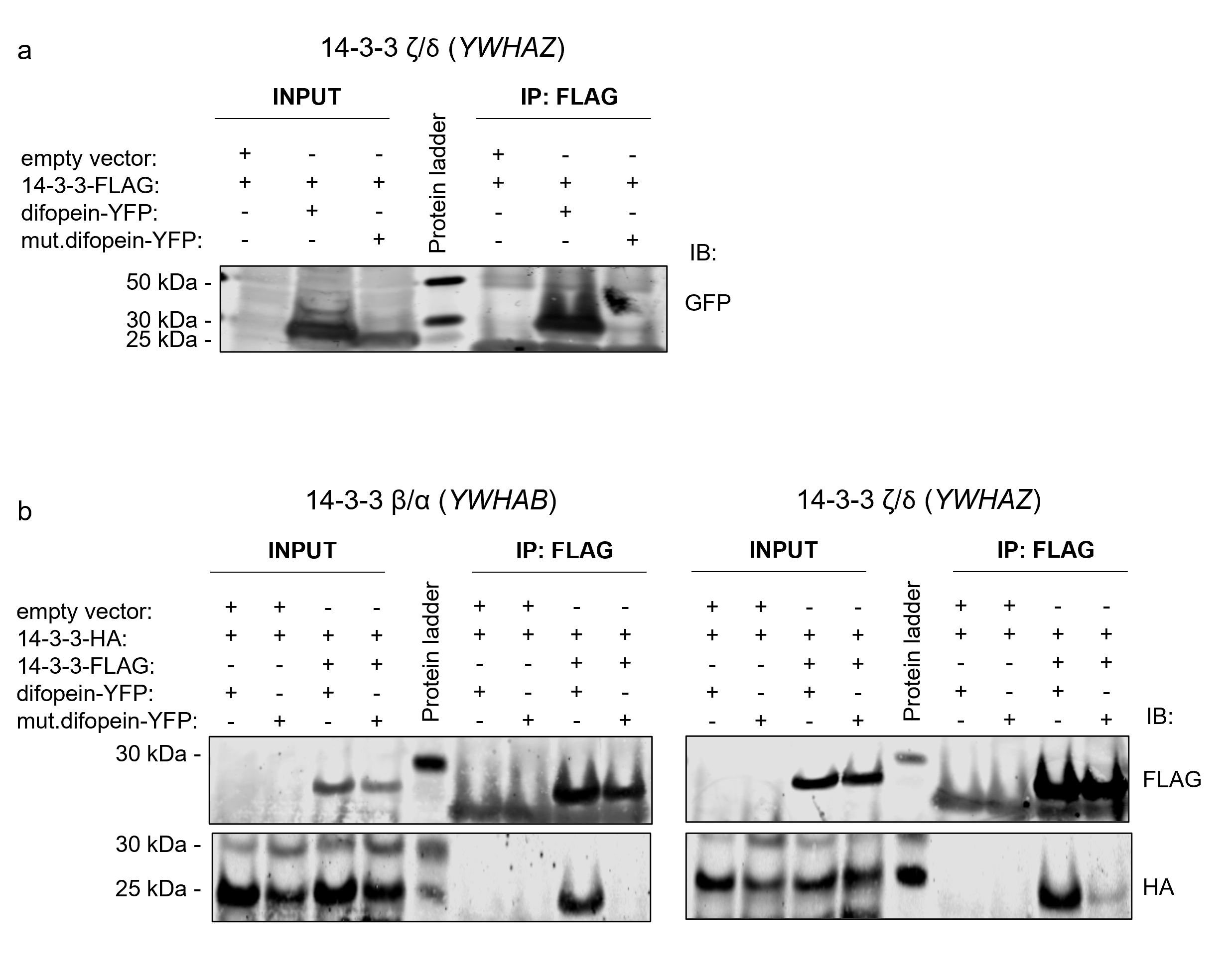

Supplement: S4 Fig — Co-immunoprecipitation analysis was performed 48 hours after transient overexpression of 14-3-3 in tsA201 WT cells. (a) Difopein strongly co-immunoprecipitated with 14-3-3 proteins, while the mutant of difopein did not. YFP-tagged difopein and its mutant were revealed with an anti-GFP antibody. (b) Difopein stabilized 14-3-3 dimers occupying its binding grooves, preventing 14-3-3 interactions with other ligands. The mutant of difopein did not stabilize 14-3-3 dimers and hence could be used as the closest relevant control for experiments involving difopein. (TIF) [file pone.0298820.s004.tif]

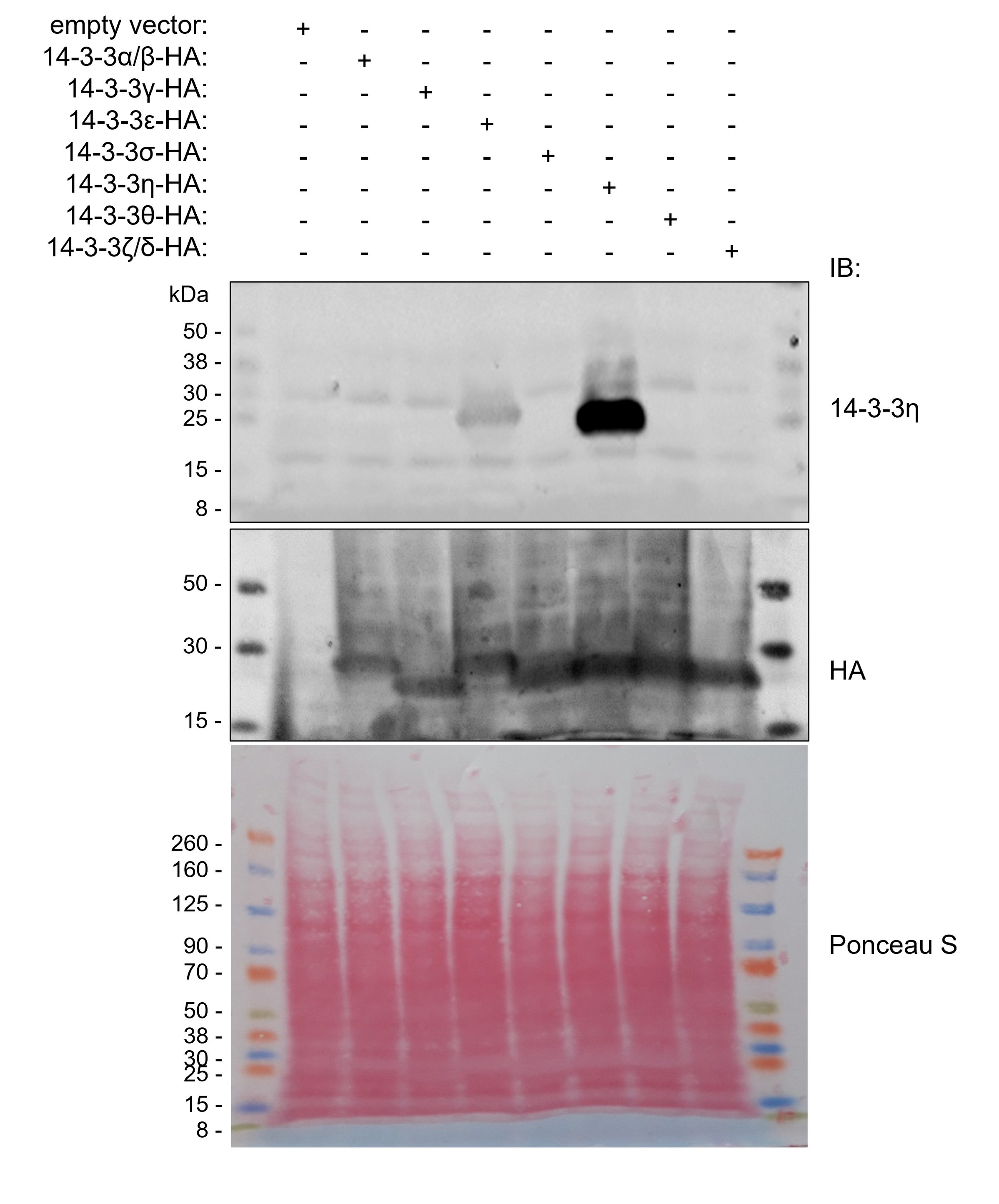

Supplement: S5 Fig — Immunoblotting analysis was performed 48 hours after transient overexpression of an empty vector or 14-3-3-HA proteins in tsA201 WT cells. Anti-14-3-3η antibody specifically detected overexpressed 14-3-3η and to a much lower extent 14-3-3ε. However, no noticeable level of endogenous 14-3-3η was detected in tsA201 transfected with an empty vector. The successful overexpression of all seven mammalian 14-3-3 isoforms was revealed with an anti-HA antibody. Ponceau S staining was performed to verify equal protein loading. (TIF) [file pone.0298820.s005.tif]
