# Supplementary material for: The dispensability of 14-3-3 proteins for the regulation of human cardiac sodium channel Nav1.5
Source: PLoS One. 2024 Mar 7;19(3):e0298820. doi: 10.1371/journal.pone.0298820 (PMC10919853; doi:10.1371/journal.pone.0298820)
Supplement: Attachment — Submitted filename: Response to Reviewers.docx [file pone.0298820.s006.docx]

Manuscript PONE-D-23-21712

Response to Reviewers

Dear Dr. Li Zhiming,

Thank you for giving us the opportunity to submit a revised version of the manuscript “The dispensability of 14-3-3 proteins for the regulation of human cardiac sodium channel Na_v_1.5” for publication in *PLOS ONE*. We appreciate the time and effort that you and reviewers dedicated to our manuscript and are grateful for the valuable comments and suggestions. We have incorporated most of the suggestions made by reviewers. Those changes are highlighted in “Revised Manuscript with Track Changes” file. Please see below, in blue, our point-by-point response to the reviewers’ comments. All line numbers refer to the “Revised Manuscript with Track Changes” file.

Reviewer #1: Iamshanova et al. investigate Nav1.5 regulation by 14-3-3 proteins in tsA201 cells. Endogenous mRNAs for all seven 14-3-3 isoforms were detectable in this cell line. Out of those, only one, 14-3-3η, was found to reliably and reproducibly interact with Nav1.5 in co-immunoprecipitation experiments. However, this interaction
appeared to be weak and/or transient. Further, analysis of immunoblots showed that both total and cell surface Nav1.5 expression remained unchanged upon overexpression of each individual 14-3-3 isoform compared to the sodium channel alone. Subsequently, difopein and a difopein mutant were used as competitors for 14-3-3/ligand interactions. Co-immunoprecipitation experiments showed 14-3-3 dimers strongly interacting with and being stabilized by difopein but not its mutant, independent of 14-3-3 isoform. Neither difopein nor its mutant affected total or cell surface Nav1.5 expression. To investigate any sodium current effects, 14-3-3η was overexpressed with and without co-expressed SCN1B and whole-cell patch clamp data was collected. Neither 14-3-3η nor SCN1B, alone or in combination, were found to modify baseline whole-cell properties of the sodium current. Difopein also failed to
alter these properties. Consistent with previous studies, further co-immunoprecipitation analysis confirmed the presence of Nav1.5-Nav1.5 interactions, but these were unaffected by the overexpression difopein. The researchers concluded that 14-3-3 proteins do not regulate Nav1.5 expression, cell surface density, or channel
function in tsA201 cells. This manuscript provides interesting data suggesting that, at least in tsA201 cells, 14-3-3 proteins do not regulate Nav1.5 to the extent previously described in older studies. Even if these results are due entirely to the differences in
cell lines used, they can still provide important groundwork for further investigations into a potentially complex and expression-system-dependent regulatory mechanism for Nav1.5. Overall, the manuscript is well written and thorough, but still has some room for improvement, as mentioned in the following critiques.

We thank Reviewer #1 for their thorough feedback.

Critiques:

1. In the “Impact of 14-3-3 proteins onto Nav1.5-mediated sodium current” section, it was found that expression of SCN1B by itself had no effect on the current properties of Nav1.5. However, no information is given regarding any potential presence of endogenous sodium channel beta subunits in this expression system. The lack of effect could be the result of endogenous beta subunits filling the role of SCN1B when it is not expressed.

We think this is an excellent suggestion. In the previous work performed by our group, we reported about the lack of endogenous Na_v_β-subunits for HEK293 cells (Laedermann *et al.*, 2013, *Front Cell Neurosci,* <https://doi.org/10.3389/fncel.2013.00137>). Nevertheless, we have also performed immunoblotting against Na_v_β1, Na_v_β2, Na_v_β3 and Na_v_β4 in our tsA201 cells and did not detect their noticeable endogenous expression levels. Please see the image below. Please note that these are unpublished data, and that we are planning to submit them for another publication.

2. Figure 4 appears to show slight but statistically insignificant effects on peak current and SSI. The authors may want to acknowledge this, while still specifying that these effects are not statistically significant. Such a small effect could be consistent with the very weak association seen between 14-3-3η and Nav1.5 in the coimmunoprecipitation experiments. Additionally, the representative traces chosen appear to show “CD8 + difopein” inactivating slightly faster than the other two conditions. If this is not generally the case, the authors may want to use alternative traces. Further, it may be worthwhile to include the inactivation time constants, as well as curves showing the recovery from inactivation.

As suggested by the reviewer we have acknowledged these slight effects described in Figure 4.

*Lines 329-330 in “Revised Manuscript with Track Changes” file: “…current properties of Na_v_1.5, besides the slight but statistically insignificant effects on the peak current density and SSI (Fig 4, Table 5).”*

We have also changed the representative traces in Figure 4b for “CD8 + empty vector” and “CD8 + mutant difopein”.

Additionally, we have calculated inactivation time constants – 𝜏_fast_ and 𝜏_slow_ (both included in excel and graphpad prism files deposited in Zenodo). Nevertheless, 𝜏_slow_ appeared to be highly variable and hence difficult to interpret. Thus, only 𝜏_fast_ was added in the main Figure 4 as panel 4e. The same calculations were performed for Figure 3 and 𝜏_fast_ was added as panel 3e. Since in both cases, Figure 3e and Figure 4e, 𝜏_fast_ did not differ within the tested conditions, we did not perform the recovery from inactivation protocol.

*Lines 218-220 in “Revised Manuscript with Track Changes” file: “Time constants of fast (𝜏_fast_) and slow inactivation (𝜏_slow_) were extracted from fitting onset of current during the step pulse with a two-component exponential according to f(t)=*$\sum_{i=1}^{n} ($*A_i_1·e −t/𝜏_i_)+C.”*

*Lines 336-337 in “Revised Manuscript with Track Changes” file: “(e) Fast inactivation constant (𝜏_fast_) plotted as a function of test potential. (f) Reversal potential (Vrev). (g) Half-voltage of SSI (V1/2 SSI). (h) Half-voltage of A…”*

*Line 340 in “Revised Manuscript with Track Changes” file: “ …* *in (c, f, g, and h)…”*

*Lines 353-355 in “Revised Manuscript with Track Changes” file: “(e) Fast inactivation constant (𝜏_fast_) plotted as a function of test potential. (f) Reversal potential (V_rev_). (g) Half-voltage of SSI (V_1/2_ SSI). (h) Half-voltage of A…”*

*Line 358 in “Revised Manuscript with Track Changes” file: “ …* *in (c, f, g, and h)…”*

*Table 4 and Table 5 were also modified by addition of 𝜏_fast_* values *at -15 mV.*

3. In Figure 3, the representative trace matched by color to the IV curve for “CD8 + hNavB1 + empty vector” seems to be mislabeled as “CD8 + mutant difopein.

Thank you for pointing this out! We have corrected the mislabelled trace in Figure 3b.

Reviewer #2: Iamshanova et al have undertaken a combination of biochemical and biophysical analyses to investigate the interaction between the human Nav1.5 protein and the 14-3-3 proteins in a heterologous cell expression system. The rationale for assessing this interaction derives from prior work demonstrating a direct interaction between 14-3-3 eta and Nav1.5, and complementary work suggesting Nav1.5 is functionally regulated by 14-3-3 proteins. However, the latter study did not establish which isoform is responsible. In the present study the authors demonstrate that of the seven isoforms only 14-3-3 eta forms macromolecular complexes with Nav1.5. This
corroborates the original study, however the relative pull down of 14-3-3 eta is still limited, as acknowledged by the authors. Further, the authors observe no functional consequence of this interaction.

We thank Reviewer #2 for their time and efforts in providing valuable and constructive feedback.

MAJOR
There are some issues with respect to the quality of the Co-IP and western blots, and the corresponding explanations within the main body text. These are detailed below:

Fig 1.
The blots for the IP have very high background. In both the Nav1.5 and HA (14-3-3) blots there are strong smears running down the borders of all 3 lanes (this does not appear to be an issue for α1-syntrophin or α-actin). As such the band highlighted by the yellow triangle is faint relative to these smears. The relative signal intensity of the two HA tagged 14-3-3 bands in the input fraction do not suggest equal expression (actin loading control suggests consistent loading). 14-3-3 eta has a visibly higher signal intensity than 14-3-3 sigma.

We agree with the Reviewer regarding the smear issue. Therefore, we have included the uncropped images of the corresponding blots. Indeed, in IP and UNBOUND fractions the migration border did not fully exit from the gel, which created additional smeary background during the revelation of 14-3-3 proteins at ≈25 kDa. And this was not the case for INPUT fraction, where migration border was nearly not present.

We agree with the Reviewer regarding the differences in the expression level of 14-3-3σ and 14-3-3η. According to the loading controls, i.e. total protein amount revealed with Ponceau S staining of the membrane as well as quantities of α1-syntrophin and α-actin, the amount of protein loaded was equal between different conditions. Thus, the difference does not arise from the loading of the gel, but rather from the level of expression. We routinely perform transfections with equal amounts of DNA within the compared conditions. Therefore, we can suggest that the difference between the expression of 14-3-3σ and 14-3-3η comes from their preferential expression in our cell model, but we cannot overspeculate about it as it was not specifically addressed in our study.

In addition to this, it looks as though there may be two bands present in the input fraction. A dense lower band and a relatively faint upper band (at least for 14-3-3 eta). Does the larger molecular weight band correspond to the immunoprecipitated fraction? If so, does the relatively weak signal in 14-3-3 sigma potentially preclude any successful IP (does a longer exposure or increased protein loading overcome this?). Do the authors have any suggestion as to the identity of the two migratory bands and why only one, notably the weaker of the two, is precipitated.

According to our experience, only one single band corresponding to 14-3-3 proteins is typically observed under the performed conditions. Thus, any faint bands were considered as aspecific background and not taken into consideration.

The identity of the bands in the Nav1.5 blots is unclear. The input lanes show a predominant band approximating 205 kDa (smaller than the molecular weight of Nav1.5) together with a faint larger migratory band. In the IP fraction the 205 kDa band is most abundant and an extra intermediate molecular weight band is also present (3 bands in total). What do the different migratory forms correspond to? (These multiple bands are not present in supplementary figs S1 or S2). Which have been included in the total Nav1.5 quantification?

In Figure 1 we used home-made 4-12% Tris-Acetate gradient gels. Under these conditions the highest molecular weight band of PageRuler™ Plus Prestained Protein Ladder migrates as 205 kDa. Predicted size of Na_v_1.5 is ≈220 kDa, which according to our immunoblotting experiment is the predominant band located around 205 kDa. From experiments when running Na_v_1.5-containing protein lysates along with Invitrogen™ HiMark™ Pre-stained Protein Standard and PageRuler™ Plus Prestained Protein Ladder we have confirmed that this predominant Na_v_1.5-specific band lays between 238 kDa and 171 kDa and a bit higher than 205 kDa, whereas the other fainter Na_v_1.5-specific band with higher molecular weight is around 460 kDa, which might correspond to the dimer of Na_v_1.5. Please see the image below. Please note that these are unpublished data, and that we are planning to submit them for another publication.

Regarding the identity of the third intermediate band in IP fraction, we cannot overspeculate, since typically we do not observe this band.

Nevertheless, the quantification of the predominant ≈205 kDa Na_v_1.5-specific band was performed throughout this study. This detail has been added to the Methods and Materials section.

*Lines 184-186 in “Revised Manuscript with Track Changes” file: “For the analysis of Na_v_1.5 protein level, the predominant band around 205 kDa was quantified.”*

Are you sure that 14-3-3 η is not interacting with the Sepharose-anti-Nav1.5 coupled beads directly? (similar to what is being seen with the FLAG beads in figs S1 and S2?)

Thank you for pointing this out! We have performed the additional control experiments similar as in S1 and S2 Figures and also observed aspecific binding of 14-3-3 proteins to Sepharose beads, especially those coupled with the antibody. Please see the image below that was added as Figure 1b.

Due to high concerns that observed phenomenon only occurs in our hands, we have performed the literature search. And it seems that such aspecificity of 14-3-3 proteins during co-immunoprecipitation experiments is rather a common issue as it was also observed by other groups. For example, in 2021 Lee *et al*., published in *PLOS ONE* how 14-3-3 proteins promote synaptic localization of NMDARs (<https://doi.org/10.1371/journal.pone.0261791>). There, in S1 Figure authors show weak but obvious band for 14-3-3 protein in control condition when immunoprecipitating IgG (pone.0261791.s001.tif). Similarly, in S1 Fig-5 (pone.0261791.s003.tif) weak but not negligible band corresponding for 14-3-3 protein was observed in control conditions immunoprecipitating mCherry IgG in both WT mice and 14-3-3 FKO mice. Please see the images below that were retrieved at <https://journals.plos.org/plosone/article?id=10.1371/journal.pone.0261791#sec018>.

S1 Figure from <https://doi.org/10.1371/journal.pone.0261791>.

S1 Fig-5 from <https://doi.org/10.1371/journal.pone.0261791>.

Overall, we thank Reviewer for their assessment. Accordingly, following changes were applied throughout the manuscript.

*Lines 257-263 in “Revised Manuscript with Track Changes” file: “…(Fig. 1a). However, the control experiment with sepharose beads on protein lysates from tsA201 cells lacking Na_v_1.5 expression also revealed aspecific bands corresponding to 14-3-3η in immunoprecipitated fractions with both immunoglobulin G coupled beads and anti-Na_v_1.5 antibody coupled beads (Fig. 1b). Therefore, these results should be interpreted with high precaution. In conclusion, due to the overall weakness of Na_v_1.5-immunoprecipitation with 14-3-3η protein, we suggest that if the interaction between Na_v_1.5 and 14-3-3η genuinely occurs it is rather weak and/or transient.”*

*Line 264 in “Revised Manuscript with Track Changes” file: “Fig 1. Na_v_1.5 co-immunoprecipitated with 14-3-3η. (a) Immunoblot of total lysate …”*

*Lines 267-270 in “Revised Manuscript with Track Changes” file: “Immunoprecipitation was performed with sepharose beads pre-coupled with anti-Na_v_1.5 antibody. (b) Immunoblot of the control experiment testing aspecific binding of 14-3-3η to sepharose beads pre-coupled with immunoglobulin G (IgG) and anti-Na_v_1.5 antibody.”*

Fig 2. There are now four or five bands present for Nav1.5 in the total lysate in panel a (there do not appear to be as many in panel c), while only one is present at the cell surface (the lowest migratory band).

Thank you! Indeed, in this particular immunoblot of total lysate many aspecific bands were detected when revealing with Na_v_1.5 antibody. However, as mentioned before only the predominant lowest band was taken into account.

Lines 243-246. “From all the isoforms, only 14-3-3η exhibited reproducibly brighter band intensity in the FLAGimmunoprecipitated fraction compared to the non-tagged fraction; hence, we concluded that only this isoform reliably interacts with Nav1.5.” There should not be any 14-3-3 protein in the immunoprecipitated fraction in the absence of a FLAG tagged protein. To suggest the relative intensity is important here is dubious given the relative intensity of the input fractions where 14-3-3 η is the most abundant. Additionally, it is not clear what has been normalised in the graph in fig S2, or how this has been achieved. The signal of 14-3-3 η bands in the input fraction is almost certainly saturated. Clarify ‘thoroughly washing the beads with TBS’ particularly given the non-specific interactions of 14-3-3 with the FLAG beads in the supplementary figures.

We agree with the Reviewer’s assessment that there should not be any 14-3-3 protein in the immunoprecipitated fraction in the absence of the FLAG-tagged protein. However, we did consistently observe a faint 14-3-3 band in IP:FLAG fraction where non-tagged Na_v_1.5 was expressed. And 14-3-3 band was much brighter in IP:FLAG fraction with the expression of FLAG-Na_v_1.5. Therefore, we reported it accordingly. Please see the images of three biological repeats of the experiment below.

Regarding the signal saturation, we confirm using the high exposure in order to visualize all the bands in INPUT and IP:FLAG fractions. Nevertheless, it was not saturated. This image was visualized with LiCor Odyssey Infrared imaging system (LI-COR Biosciences) that detects the saturated signals automatically marking them as white spots and we do not have possibility to tamper with them. For example, the saturated signal is clearly visible at the 50 kDa band in the protein ladder. Please see the image of the uncropped immunoblot below.

To clarify the ‘thoroughly washing the beads with TBS’ we applied following changes:

*Lines 155-156 in “Revised Manuscript with Track Changes” file: “…and were mixed with beads that were washed three times with TBS and then coupled with antibody at 4°C overnight. After six times washing the beads with TBS, the co-immunoprecipitated protein complex was eluted…”
Line 157 in “Revised Manuscript with Track Changes” file: “After washing the beads with TBS for six times, …”*

I am not convinced that the appropriate statistical analysis has been carried out for the data in figure 2. I appreciate in the methods it is stated that when the data is normalised to the control condition a t-test has been used. But all 7 different experimental samples were run together with the single control, in which you were quantifying the same protein and presumably in a linear range for densitometric analysis. So, wouldn’t a Kruskal-Wallis ANOVA (nonparametric) be more appropriate?

Thank you for raising this point. These data were whether not sufficient in quantity (n=3) to assess their normality or rather normally distributed (according to Shapiro-Wilk test). Moreover, these were normalized data, which were compared to the control condition with hypothetical value=1. Thus, we chose One Sample T-test as the most appropriate statistical test to use in this case. We believe that it would be more appropriate to use Kruskal-Wallis ANOVA when the data would not be normally distributed and/or when the control condition would be expressed as an absolute value rather than the normalized hypothetical value of 1.

Lines 291-293. Please clarify. In fig S4a the different migratory bands are not clear. What are the different molecular weights? Also, the subsequent sentence states difopein stabilises 14-3-3 dimers.

Thank you! In S4a Figure the immunoblot was revealed with antibody against GFP, which was used to visualize YFP-tagged difopein and YFP-tagged mutant of difopein. Difopein is a dimer of R18 peptide and hence is able to bind two 14-3-3 proteins. Since 14-3-3 proteins may form dimers, it was suggested that difopein would stabilize 14-3-3 dimers occluding their sites from binding to other ligands. Mutant of difopein is a single R18 peptide with mutated 14-3-3 binding site. Theoretical size of difopein is 6.36 kDa, whereas for mutant of difopein is 2.36 kDa. Since both are fused with YFP (27 kDa), their sizes should be ≈30 kDa with the mutant of difopein having 4 kDa smaller molecular weight. Such difference in the migration sizes was observed in our hands as well as by our colleagues who originally created these constructs (Masters and Fu, 2001, *JBC*; <https://doi.org/10.1074/jbc.M105971200>):

Figure 1 and Figure 5 from <https://doi.org/10.1074/jbc.M105971200>.

The monomer has a molecular weight of 30kDa, so presumably the dimer is 60kDa? Not only is this not made clear in the text, fig S4b is not labelled to highlight monomeric/dimeric bands, or even show any migration around 60 kDa. The panels display a region covering two protein ladder markers of 25 kDa and 30 kDa.

Indeed, monomeric 14-3-3 protein is ≈30 kDa. Since we did not perform native polyacrylamide gel electrophoresis, but rather classical SDS-PAGE we did not expect and did not observe any 14-3-3 dimers or their complexes with difopein. The aim of this co-immunoprecipitation experiment in S4a Figure was to validate that 14-3-3 efficiently binds to difopein, but not to the mutant of difopein. Please see the uncropped immunoblot below.

It is unclear why these supplementary experiments did not include the only isoform (14-3-3 eta) that has been demonstrated to interact with Nav1.5.

The aim of the experiment in S4b Figure was to validate that difopein has ability of binding two 14-3-3 proteins as previously described in the literature. Since different combinations between various 14-3-3 proteins are possible within the heterodimer and their pairing preference is not yet clear, we have chosen to rather test homodimers of 14-3-3 proteins. At this point, we had all seven 14-3-3 proteins tagged with HA, but only 14-3-3β/α and 14-3-3ζ/δ tagged with FLAG. Therefore, for validation of difopein as a high affinity molecule with the homodimers of 14-3-3 proteins, we used tools available to us at the time - 14-3-3β/α-HA with 14-3-3β/α-FLAG as well as 14-3-3ζ/δ-HA with 14-3-3ζ/δ-FLAG. In S4b Figure we show that immunoprecipitating 14-3-3-FLAG leads to significant co-immunoprecipitation with 14-3-3-HA in the presence of difopein, but not in the presence of the mutant of difopein.

The whole-cell voltage clamp raw data are of high quality. A minor point, in Fig 4, the representative current waveforms in panels b for CD8 + empty vector do not appear to correspond with the IV curve. The last current waveform displayed peaks around -50 pA/pF, yet on the IV curve it is almost at 0 pA/pF. Are some currents missing from the representative plot in panel B, or does the voltage-dependence of this particular data set differ from that represented in panel A?

Thank you! We have changed the representative traces in Figure 4b for “CD8 + empty vector” and “CD8 + mutant difopein”.

Although the raw data is of high quality, there is a noticeable variability in some key parameters, and an unusually high n number. Panel e, a significant difference (P=0.0161) is highlighted between CD8 + empty vector and CD8 +difopein. No explanation has been given for this variation in reversal potential. It is also peculiar that there is such a large scatter of the Vrev, particularly for CD8 + empty vector, with values ranging from 20 mV up to 60 or 65 mV. Taking into account the reported internal and external Na+ concentrations in panel a, the equilibrium potential should of the order of 26 mV. Do the currents that strongly deviate (i.e. those around 60 mV) also have V1/2s that deviate from the mean? I note that the authors have commented on the polyclonal nature of their cell line as being implicated in the variability of Nav1.5 expression - this could account for variations such as current density, but not reversal potential.

Thank you for pointing this out! Indeed, in Figure 4e, the *V*_rev_ between “CD8 + empty vector” appeared to be significantly different from “CD8 + difopein” condition (*p*=0.0161). However, when comparing “CD8 + difopein" with its more relevant control condition, i.e. “CD8 + mutant of difopein”, the statistical significance was absent (*p*=0.3628). Therefore, we cannot conclude that the effect observed on *V*_rev_ is due to the difopein directly and not due to other artifacts, e.g. specificity of the vector, presence of YFP tag and etc.

We did not compensate for the liquid junction potential in our study (already mentioned in Materials and Methods section). It could explain why the mean *V*_rev_ values are around 40 mV rather than the theoretically expected 26 mV. And yes, the currents from 3 cells in “CD8 + empty vector” condition with high *V*_rev_ (around 60 mV) also have the highest *V*_1/2_ of activation and inactivation. Our criteria for exclusion of cells from analysis were (already described in Materials and Methods section): the absence of *I*_Na_; current at holding potential (-100 mV) <= -100 pA; slope of the activation (A) curve <=4; and slope of the steady-state inactivation (SSI) curve >=8. Since these 3 cells were within the aforementioned criteria, they were not excluded.

Line 421 – 425. Why does co-expressing SCN1B eliminate the possibility that the discrepancy could be due to the presence of the NavB1 subunit? Is this referring to endogenous expression? Which experiments was SCN1B coexpressed in? The methods are not clear.

Thank you! In the previous sentence (lines 418-420), we were describing the work of Allouis *et al.*, who showed that 14-3-3η shifted the inactivation curve of Na_v_1.5-medaited current. However, in their study they performed co-expression with Na_v_β1 subunit. Therefore, to eliminate the discrepancy between their work and our study we also included condition when Na_v_β1 was co-expressed. To clarify this for the readers we applied following changes:

*Lines 440-441 in “Revised Manuscript with Track Changes” file: “By co-expressing SCN1B, we eliminated the possibility that such discrepancy between our studies could be due to the presence of the Na_v_β1 subunit.”*

MINOR
In Fig 2, I think the 205 kDa label is in the wrong location on panel a. Additionally, the data in the graphs in panel b occupy less than 50% of the graphical space. Are 4 significant figures necessary for the P values presented?

Thank you for spotting it! We have corrected the labelling in Figure 2a. If needed, we can move Figure 2b and Figure 2d in the Supplementary Figures. But we believe that it is easier for the readers to have representative immunoblots alongside with their corresponding quantified data.

Line 44 – grammatical error, either a comma or ‘the’ is missing between heart and voltage-gated.

Thank you!

*Line 44 in “Revised Manuscript with Track Changes” file: “In the heart, the voltage-gated sodium channel, Na_v_1.5 (encoded by SCN5A gene), …”*

Line 124 and Line 139 - Cell pellets or cell monolayers are referred to as ‘prewashed (PBS)’ what does this mean? Cells were washed 'x' times in PBS?

*Lines 124-127 in “Revised Manuscript with Track Changes” file: “**Cell monolayers were once washed with PBS, detached with 0.05% Trypsin-EDTA, and pelleted by centrifugation at 200 rpm for 5 minutes. Afterwards, cell pellets were washed with PBS three times more and then taken for RNA extraction using ReliaPrep^TM^ RNA cell Miniprep Systems according to the manufacturer’s instruction (Z6010, Promega).”*

Line 149 – typo? ‘nProtein A Sepharose’
*Line 151 in “Revised Manuscript with Track Changes” file: “nProtein**^TM^ A Sepharose”*

Lines 152 -154 Clarify ‘prewashed’ and ‘thoroughly washing’ (this wording occurs again at line 164, and also needs clarification).

*Lines 155-157 in “Revised Manuscript with Track Changes” file: “…and were mixed* *with beads that were washed three times with TBS and then coupled with antibody at 4°C overnight. After washing the beads with TBS for at least six times, …”*

Lines 366-370. The luminescent signal intensity in Fig 5 is of a similar order of magnitude for d, f and g. Only e (N-C) has a lower signal intensity. So the conclusion that N-N and C-C termini combinations were brightest compared to N-C and C-N is not entirely accurate.

We agree with the Reviewer’s assessment. Accordingly, we have revised the manuscript as follows.

*Lines 387-389 in “Revised Manuscript with Track Changes” file: “…luminescent signal was detected when N-N termini were combined, compared to the N-C, C-N, and C-C conditions (Figs 5d-g).”*

Line 435 – 439. Ref 14 is Clatot et al. They did observe 14-3-3 participating in dimerization and did not specifically assess 14-3-3 η.

While we appreciate the Reviewer’s feedback, we respectfully disagree. Reference 14 is Clatot *et al.*, 2017, Nat Commun; <https://www.nature.com/articles/s41467-017-02262-0>. In the section Result “DN effect abolished by 14-3-3 inhibition” on page 6, the authors state the following: “… 14-3-3 does not mediate the direct dimerization of sodium channels even though it is implicated in the DN mechanism…”. Furthermore, in the section Discussion on page 10, they write: “As observed from the co-immunoprecipitations in Fig. 2c, d, the first interaction site of 14-3-3 is lost but the interaction between α-subunits remains. This suggests that the dimerization site between α-subunits is independent of 14-3-3 interaction.” Please see the printscreens of the aforementioned citations below.

Therefore, we are steadfast that Clatot *et al.*, 2017, Nat Commun; concluded that 14-3-3 proteins do not mediate the dimerization of Na_v_1.5, which is corroborated by our current study.

From Clatot *et al.*, 2017, Nat Commun (<https://www.nature.com/articles/s41467-017-02262-0>)

Reviewer #3: It is now fairly clear that sodium channels such as Nav1.5 can interact on the plasma membrane as dimers. In 2017, Clatot et al reported that 14-3-3 mediated this interaction. The current paper addresses the necesssity of 14-3-3 in this process. Although the authors show a weak interaction between 14-3-3? (but no other
14-3-3 isoforms) and Nav1.5, they find no evidence that 14-3-3 is required for Nav1.5 dimerisation; neither can they find evidence for any functional requirement of 14-3-3 in Nav1.5 dimerisation or activity. If correct, this is important, as on the face of it, the data is not consistent with outher work on the same question.

We thank Reviewer #3 for their insightful feedback, but we respectfully disagree.

Reference 12 is Allouis *et al.*, 2006, *Circ Res*, (<https://doi.org/10.1161/01.RES.0000229244.97497.2c>), who in accordance with our findings showed that exogenous 14-3-3η did not modify *I*_Na_ density: “The presence of overexpressed human 14-3-3η (h14-3-3η) in COS-7 cells expressing hNav1.5 and hβ1 subunits, did not modify the Na^+^ current density…”

Reference 14 is Clatot *et al.*, 2017, Nat Commun (<https://www.nature.com/articles/s41467-017-02262-0>), who similarly to us concluded that dimerization between α-subunits is independent of 14-3-3 interaction: “… 14-3-3 does not mediate the direct dimerization of sodium channels even though it is implicated in the DN mechanism… This suggests that the dimerization site between α-subunits is independent of 14-3-3 interaction.”

Reference 22 is Utrila *et al.*, 2018, Front Physiol (<https://www.frontiersin.org/articles/10.3389/fphys.2017.00903/full>), who reported that inhibition of 14-3-3 proteins did not modify the *I_Na_,* which was also corroborated by our current electrophysiological findings with difopein.

Therefore, at least 3 previous outer works are in accordance with our data.

The fundamental problem is that the cells used in these experiments express endogenous 14-3-3 isoform, including the η isoform (see Sup Fig 3). This means that any signal generated by overexpressing 14-3-3 isoforms may be small. This may for example, explain why the abslolute amount of 14-3-3η immunoprecipitated with Nav1.5 (Fig 1) is weak. The authors use difopein (an inhibitor of 14-3-3 dimerisation) to show that 14-3-3 dimerisation does not affect Nav1.5 dimerisation. But it is possible that 14-3-3 is required for the initial assembly of the Nav1.5 dimers, rather than a continuous requirment. This is why knocking down 14-3-3? by for example, RNAi, is required to really rule out an role in Nav1.5 dimerisation in this particular experimental system.

Thank you for this suggestion. The levels of mRNA do not necessarily correlate with the level of protein expression. Nevertheless, we agree with the Reviewer’s assessment that the presence of endogenous 14-3-3η may represent potential limitation of the study. To address it, we have ordered antibody specific against 14-3-3η (abcam, ab206292) and verified its specificity. After transfecting tsA201 WT cells with an empty vector or all 14-3-3 proteins separately, we confirmed that this antibody successfully recognizes 14-3-3η and to a much lower extent 14-3-3ε. However, no noticeable level of endogenous 14-3-3η was detected in tsA201 transfected with an empty vector. Please see the image below that was added to the current work as S5 Figure. Furthermore, as suggested by Reviewer, we performed silencing of endogenous 14-3-3η in our cell model. For this, we have purchased siRNA targeting *YWHAH* (Ambion, s14967). Transfection was performed by using Lipofectamine™ RNAiMAX Transfection Reagent (Invitrogen, 13778075) according to the manufacturer’s instructions. 48 hours after transfection, cells were lysed and analysed by SDS-PAGE. Protein lysate of tsA201 cells overexpressing 14-3-3η-HA was run alongside with the siRNA lysates as a positive control for 14-3-3η band. No obvious band for 14-3-3η was detected in both conditions siCTRL and siYWHAH. Thus, we could not proceed with silencing of endogenous 14-3-3η further. Please see the image below.

Overall, we suggest that the endogenous level of 14-3-3η is rather low and is unlikely to compete with the overexpressed 14-3-3η-HA, especially since Na_v_1.5 is also overexpressed in our study. Therefore, following modifications were applied:

*Lines 487-493 in “Revised Manuscript with Track Changes” file: “To challenge this limitation, we compared endogenous and exogenous protein levels of 14-3-3η in our cell model. No noticeable level of endogenous 14-3-3η was detected when comparing it to the level of heterologously overexpressed 14-3-3η-HA (S5 Fig). Thus, we suggest that the endogenous level of 14-3-3η is rather low and is unlikely to compete with the overexpressed 14-3-3η-HA, especially since Na_v_1.5 has also been overexpressed in our study.”*

*Lines 697-704 in “Revised Manuscript with Track Changes” file: “S5 Fig. Investigation of endogenous 14-3-3η protein level in tsA201. Immunoblotting analysis was performed 48 hours after transient overexpression of an empty vector or 14-3-3-HA proteins in tsA201 WT cells. Anti-14-3-3η antibody specifically detected overexpressed 14-3-3η and to a much lower extent 14-3-3ε. However, no noticeable level of endogenous 14-3-3η was detected in tsA201 transfected with an empty vector. The successful overexpression of all seven mammalian 14-3-3 isoforms was revealed with an anti-HA antibody. Ponceau S staining was performed to verify equal protein loading.”*

Some additional points:
Figure 1: the 14-3-3 band in the IP fraction (indicated by the yellow arrow head) has a noticable runs noticably higher than the 14-3-3 protein in the input. But interestingly, the input band does have a fainter, higher band that could be the 14-3-3 in the IP fraction. Does this indicate a small fraction of modified (eg phosphorylation?) 14-3-3?

Thank you! It is difficult to compare such small differences in the molecular weights, especially since the migration of the gel could sometimes deviate from the straight line and the images might be slightly tilted when taken. Therefore, we cannot overinterpret the identity of the fainter higher band. And to suggest that it could be phosphorylated form of 14-3-3 further experiments are required, which is beyond the scope of the current study.

The authors say "..when comparing various locations of LgBiT and SmBiT tags on Nav1.5, the brightest luminescent signal was detected when N-N and C-C termini were combined, compared to the n-C and C-N conditions (Figs 5d-g)". This statement may be true fo N-C, but C-N doesn't look much different from N-N .

Thank you! N-N signals were consistently high within technical and biological repeats, whereas C-N signals displayed much larger variability.

*Lines 387-389 in “Revised Manuscript with Track Changes” file: “…luminescent signal was detected when N-N termini were combined, compared to the N-C, C-N, and C-C conditions (Figs 5d-g).”*
