# Supplementary material for: The dispensability of 14-3-3 proteins for the regulation of human cardiac sodium channel Nav1.5
Source: PLoS One. 2024 Mar 7;19(3):e0298820. doi: 10.1371/journal.pone.0298820 (PMC10919853; doi:10.1371/journal.pone.0298820)
Supplement: Attachment — Submitted filename: Response to Reviewers 240124.docx [file pone.0298820.s007.docx]

Manuscript PONE-D-23-21712R1

Response to Reviewers

Dear Dr. Li Zhiming,

Thank you for giving us the opportunity to submit the second revised version of the manuscript “The dispensability of 14-3-3 proteins for the regulation of human cardiac sodium channel Na_v_1.5” for publication in *PLOS ONE*. We appreciate the time and effort that you and reviewers dedicated to our manuscript and are grateful for the valuable comments and suggestions. We have accepted all the changes that were previously suggested by the reviewers and have now incorporated most of the suggestions made by Reviewer #2. Those changes are highlighted in the updated “Revised Manuscript with Track Changes 240124” file. Please see below, our point-by-point response to the reviewers’ comments. All line numbers refer to the updated “Revised Manuscript with Track Changes 240124” file.

Reviewer #2: The revisions and added experiments strengthen the authors findings and sufficiently address my comments. I apologise for the confusion regarding the point of the role of 14-3-3 in Nav1.5 dimerization. The authors are correct 14-3-3 is not necessary for dimerization, as observed here and in prior studies, although it could possibly further stabilise the complex. In contrast with others, no functional effects were observed leading the authors to conclude that 14-3-3 proteins do not regulate Nav1.5 in tsa201 cells (the model system perhaps accounting for the variability).

Response: Thank you! We greatly appreciate Reviewer’s openness and constructive feedback.

Remaining points:

Lines 217 – 220: Description of double exponential function has presumably omitted the word ‘decay’ or ‘inactivation’ from line 218 (after current). NB. The values for tfast vary unexpectedly at -30 mV in fig4e for CD8 + difopein. The CD8 + empty vector values are surprisingly similar between -40 to -30 mV in fig 3e

Response: Thank you for spotting it! Changes were applied as follows.

*Line 228:* “.. were extracted from fitting onset of current decay ..”

Line 217 – V1/2 also applies to inactivation.

Lines 213-223: “.., where *y* is the normalized peak current (pA/pF) at a given holding potential, *g* is the maximal conductance, *V_m_* is the membrane potential, *V_rev_* is the reversal potential for Na^+^, *V_1/2_* is the potential at which half of the channels are activated and *k* is the slope factor of activation. Activation (A) curves were fitted with the Boltzmann equation *y = 1-(1/(1 + exp[(V_m_ – V_1/2_)/k]))*, where *y* is the normalized conductance at a given holding potential, *V_m_* is the membrane potential, *V_1/2_* is the potential at which half of the channels are activated and *k* is the slope factor of activation. SSI curves were fitted with the Boltzmann equation *y = 1/(1 + exp[(V_m_ – V_1/2_)/k])*, where y is the normalized current at a given holding potential, *V_m_* is the membrane potential, *V_1/2_* is the potential at which half of the channels inactivated and *k* is the slope factor of inactivation.”

Line 442 – 445.

“Indeed, previous studies reported conflicting results regarding the impact of Navβ1 on the function of Nav1.5. While some groups detected changes of the peak current density and/or differences in the channel kinetics and gating properties, others like us did not observe any Navβ1-mediated changes of INa [24–28].”

The effects of beta 1 are variable in the literature, but of references 24 – 28, I do not agree that any of these support an absence of any effect of beta 1 on INa.
24: Increased current density, negative shift of V1/2 activation and accelerated recovery.
25: Authors have previously shown that beta 1 causes a negative shift of Nav1.5 SSI in both oocytes and HEK293 cells (HEK293 cells; supplementary data in Zhu et al 2017 JGP). In the referenced paper (25), WT and SCN1B null myocytes are compared and peak current is smaller in the presence of beta 1.

26: Shift of voltage dependence of SSI

27: Shift of voltage dependence of SSI

28: Shift of voltage dependence of SSI

Response: Thank you for the valid comment! We have added more references into this paragraph and developed further our argumentation as follows.

Lines 454-468: “..of *I*_Na_ [24–31]. In particular, cardiomyocytes of *Scn1b* null mice exhibited increased macroscopic *I*_Na_ without any differences in voltage dependence or kinetics of the current [29]. Further investigation revealed that this *Scn1b*-dependent *I*_Na_ increase was mostly coming from the midsection of cardiomyocyte rather than the intercalated discs, it was tetrodotoxin-sensitive, and was concomitant with the increase of *Scn3a* mRNA [30]. Altogether suggesting on the unlikelihood of biophysical regulation of the native Na_v_1.5 by Na_v_β1 [30]. Another recent study demonstrated the importance of cellular model with its endogenous expression of Na_v_β-subunits when investigating the biophysical properties of Na_v_1.5 [31]. Specifically, co-expression of Na_v_β1 did not exert any effect on Na_v_1.5-mediated current in eHAP, human haploid fibroblasts-like cells with comparable endogenous mRNA levels of Na_v_β-subunits to human embryonic kidney (HEK293) cells, but significantly affected *I*_Na_ in gene-edited eHAP, where genes coding for endogenous *SCN1B-SCN4B* and their phylogenetic relatives were priorly disrupted [31].”

Line 472: “.. from HEK293 cells [36].”

References added:

Lines 655-667:

“29.	Lopez-Santiago LF, Meadows LS, Ernst SJ, Chen C, Malhotra JD, McEwen DP, et al. Sodium channel Scn1b null mice exhibit prolonged QT and RR intervals. J Mol Cell Cardiol. 2007;43: 636–647. doi:10.1016/j.yjmcc.2007.07.062

30.	Lin X, O’Malley H, Chen C, Auerbach D, Foster M, Shekhar A, et al. Scn1b deletion leads to increased tetrodotoxin-sensitive sodium current, altered intracellular calcium homeostasis and arrhythmias in murine hearts. Journal of Physiology. 2015;593: 1389–1407. doi:10.1113/jphysiol.2014.277699

31.	Minard AY, Clark CJ, Ahern CA, Piper RC. Beta-subunit-eliminated eHAP expression (BeHAPe) cells reveal subunit regulation of the cardiac voltage-gated sodium channel. Journal of Biological Chemistry. 2023;299. doi:10.1016/j.jbc.2023.105132”

Regarding the variation of vrev. I agree with the authors, the liquid junction potential likely accounts for the difference in recorded values of vrev when compared with the theoretical value, which is fine assuming it is stable. It is also highly unlikely that difopein is affecting the selective Na+ permeability of the channel pore. But, in some cells there is a large deviation of vrev. Assuming that the Na+ permeability is not affected, this variation has most likely arisen from some technical issue, for which the vector specificity and tags do not seem like a likely source.... a more likely culprit (though not necessarily the case here!) would be something like the integrity of the electrode (e.g need for re-chloridisation). If this, or some other source was identified it would be a reason to exclude the affected data points. In the absence of some identifiable reason, of course under the present exclusion criteria stated by the authors the data should be included, but perhaps commented on (assuming the authors find this variability odd too).

Response: Thank you for your valid comment! Indeed, we also found these 3 cells odd. However, when trying to identify the “culprit” of such effect based on the experimental dish, day or condition, we could not conclude on any obvious technical or biological effect that might cause such variability.

Regarding fig 2. I was not suggesting that you should remove the panels in b, just that the scale could be adjusted to fully visualise the data points. The values of your data all appear to lie in the region of ~0.5 – <2, but the scale of your y-axis extends to 4.

Response: Thank you for the clarification! We have adapted the y axes in Fig. 2b from 0 to 2.5 as kindly suggested by the Reviewer.
